# Adrenaline auto-injector prescription and patients’ administration proficiency

**DOI:** 10.1186/2045-7022-5-S3-P12

**Published:** 2015-03-30

**Authors:** Luis Amaral, Alice Coimbra, Jose Luis Placido

**Affiliations:** 1 Serviço de Imunoalergologia, Centro Hospitalar de São João E.P.E., Porto, Portugal

## Background

Adrenaline is the first-line emergency treatment for anaphylaxis. The only adrenaline auto-injector (AAI) currently available in Portugal is Anapen^®^. The aim of this study was to evaluate adrenaline prescription in the Emergency Department (ED) and the patients' capacity to correctly simulate adrenaline administration.

## Methods

Patients with food allergy (FA) and hymenoptera venom allergy (HVA) followed in our department were invited to simulate adrenaline administration with an Anapen^®^ trainer and a medical record review was performed.

## Results

A total of 36 individuals (50% male, mean age 34.5 years) were included; 28 with HVA (26 on venom immunotherapy) and 8 with FA. Twenty-seven went to an ED. Adrenaline was administered in only 3 and just5 of them were discharged with an AAI prescription. The remaining 22 had their AAI prescribed and taught how to use it, only after consultation with an Allergist. Twenty-three (64%) had their Anapen^®^ with them and 19 (53%) admitted carrying it on a daily basis. Fourteen (39%) performed the simulation correctly, although 7 of them did not massage the injection site, as instructed by the manufacturer. On average, 2 training sessions were performed per patient but, despite this, 22 (61%) failed at various steps, including 4 who injected with the wrong end. Six reported using their Anapen^®^ in a real life situation, but curiously 4 of them did not simulate the administration effectively.

## Comments

According to the WAO guidelines, patients with anaphylaxis history should carry an AAI. Adrenaline underuse and a deficient prescription of adrenaline (14%) on discharge from the ED as well in non-specialized centres was evident. Approximately two thirds of our patients failed to demonstrate proper administration, regardless of previous patient education. This is an unacceptably high proportion of at risk patients. This device requires repeated and regular training sessions since this is the only AAI available in Portugal. Adrenaline is potentially lifesaving if the patient is willing to use it and is capable of injecting it correctly. Practice makes perfect.

